# Poloxamer 188 Exerts Direct Protective Effects on Mouse Brain Microvascular Endothelial Cells in an In Vitro Traumatic Brain Injury Model

**DOI:** 10.3390/biomedicines9081043

**Published:** 2021-08-19

**Authors:** Felicia P. Lotze, Matthias L. Riess

**Affiliations:** 1 Department of Anesthesiology, Vanderbilt University Medical Center, Nashville, TN 37232, USA; felicia.lotze@gmail.com; 2 Department of Anesthesiology, University Medicine Greifswald, 17475 Greifswald, Germany; 3 Department of Pharmacology, Vanderbilt University, Nashville, TN 37232, USA; 4 Anesthesiology, TVHS VA Medical Center, Nashville, TN 37212, USA

**Keywords:** copolymer, hypoxia reoxygenation, ischemia reperfusion, neuroprotection, P188, stroke

## Abstract

Traumatic Brain Injury (TBI), the main contributor to morbidity and mortality worldwide, can disrupt the cell membrane integrity of the vascular endothelial system, endangering blood–brain barrier function and threatening cellular subsistence. Protection of the vascular endothelial system might enhance clinical outcomes after TBI. Poloxamer 188 (P188) has been shown to improve neuronal function after ischemia/reperfusion (I/R) injury as well as after TBI. We aimed to establish an in vitro compression-type TBI model, comparing mild-to-moderate and severe injury, to observe the direct effects of P188 on Mouse Brain Microvascular Endothelial Cells (MBEC). Confluent MBEC were exposed to normoxic or hypoxic conditions for either 5 or 15 h (hours). 1 h compression was added, and P188 was administered during 2 h reoxygenation. A direct effect of P188 on MBEC was tested by assessing cell number/viability, cytotoxicity/membrane damage, metabolic activity, and total nitric oxide production (tNOp). While P188 enhanced cell number/viability, metabolic activity, and tNOp, an increase in cytotoxicity/membrane damage after mild-to-moderate injury was prevented. In severely injured MBEC, P188 improved metabolic activity only. P188, present during reoxygenation, influenced MBEC function directly in simulated I/R and compression-type TBI.

## 1. Introduction

As the main contributor to morbidity and mortality, Traumatic Brain Injury (TBI) affects millions of people each year worldwide [1]. Besides mechanically harming neurons, TBI can cause disruption of the vascular endothelial system as well as cell membrane integrity [2]. Thereby, TBI endangers blood brain barrier (BBB) function and cerebral blood flow, causing ischemia, amplifying BBB damage, and ultimately threatening the survival of multiple cell types [3]. The impact injures brain tissue instantly; however, this primary injury can only be prevented by precautions [4]. Still, secondary injuries originate as the impact hits the brain and can continue for several years [2]. Thus, most research focuses on the complex mechanisms involved in secondary injury [5]. Even though the knowledge of pathomechanisms underlying secondary injuries is increasing at an enormous pace, therapies specifically targeting cell death, oxidative stress, inflammation, BBB breakdown, excitotoxicity, and edema formation are not yet fully established [2,6].

Ischemia is thought to be of major importance in secondary brain injury [7]. It occurs commonly in patients who sustained TBI and can last up to 10 days following the mechanical injury [8]. Even though it is inevitable to restore perfusion, reperfusion can aggravate the ischemic harm [9]. The resulting injury—called ischemia/reperfusion (I/R) injury—can be found in many organs, e.g., resulting from an acute coronary syndrome, stroke, carotid endarterectomy, aneurysm repair, circulatory arrest, and TBI [7].

Poloxamer 188 (P188), a non-toxic, nonionic linear synthetic triblock copolymer, is built of a hydrophobic polypropylene oxide center bordered by two hydrophilic polyethylene oxide chains [10,11,12]. P188 displayed protective effects against I/R injury as well as against injury following TBI in multiple in vitro models [13,14,15,16,17,18,19,20,21]. Simulations suggest that P188 might help to resolve the damage of the cellular membrane, by insertion into pores within the bilayer to seal membrane defects [12,22]. Besides sealing neuronal cell membranes, one major mechanism underlying neuroprotection is suggested to be the salvation of cells comprising the BBB [23,24]. Therefore, we hypothesized that part of the neuroprotective effect observed by P188 can be attributed directly to brain endothelial cell protection. Hence, we intended to:

(1) Develop a suitable in vitro compression-type TBI model using brain endothelial cells to test for potential treatments.
(2) Evaluate direct effects of P188 given upon reoxygenation in an in vitro compression-type TBI model, applying mild-to-moderate and severe injury.
(3) Assess protective effects of P188 post-treatment after excessive damage in vitro.
(4) Test for osmotic effects by using polyethylene glycol (PEG) with a similar molecular weight as P188 serving as control.

## 2. Materials and Methods

The model development was described previously in further detail [25,26,27].

### 2.1. Cell Culture

For experimental performances, isolated C57BL/6 Mouse Primary Brain Microvascular Endothelial Cells (MBEC) from Cell Biologics Inc. (Chicago, IL, USA) were obtained. MBEC were recovered from cryo-preservation and maintained following the manufacturer’s instructions. The complete mouse endothelial cell medium (500 mL, Cell Biologics Inc.) containing 0.5 mL vascular endothelial growth factor (0.1%), 0.5 mL endothelial cell growth supplement (0.1%), 0.5 mL heparin (0.1%), 0.5 mL epidermal growth factor (0.1%), 0.5 mL hydrocortisone (0.1%), 5.0 mL L-glutamine (1%), 5.0 mL antibiotic-antimycotic solution (1%) and 25 mL fetal bovine serum (FBS) was supplemented with another 25 mL FBS (Cell Biologics Inc.), increasing the concentration of FBS from 5 to 10%, and was stored at 4 °C for less than 2 months. All dishes used for cell culturing of MBEC were pre-coated with gelatin-based coating solution (Cell Biologics Inc.) directly before utilization. MBEC were plated at seeding densities of 50,000 and 75,000 cells/cm^2^. Afterward, MBEC were grown to 90–100% confluency up to 20 passages, maintaining humidified culturing conditions (21% O_2_, 5% CO_2_, 74% N_2_, 37 °C).

### 2.2. Compression-Type TBI Model

**Figure 1:**
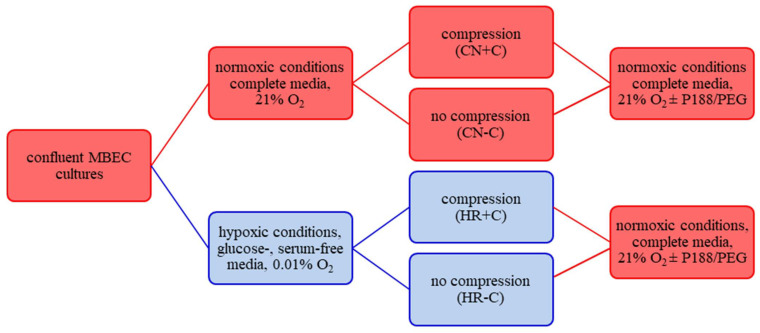
Schematic representation of the experimental setup. CN-C was used as control, while CN+C simulated contusion, HR-C represented stroke and HR+C mimicked traumatic brain injury. Abbreviations: cm, centimeter; MBEC, mouse primary brain microvascular endothelial cells; O_2_, Oxygen; P188, Poloxamer 188; PEG, polyethylene glycol; Colors: red, normoxic conditions; blue, hypoxic conditions.

**Figure 2:**
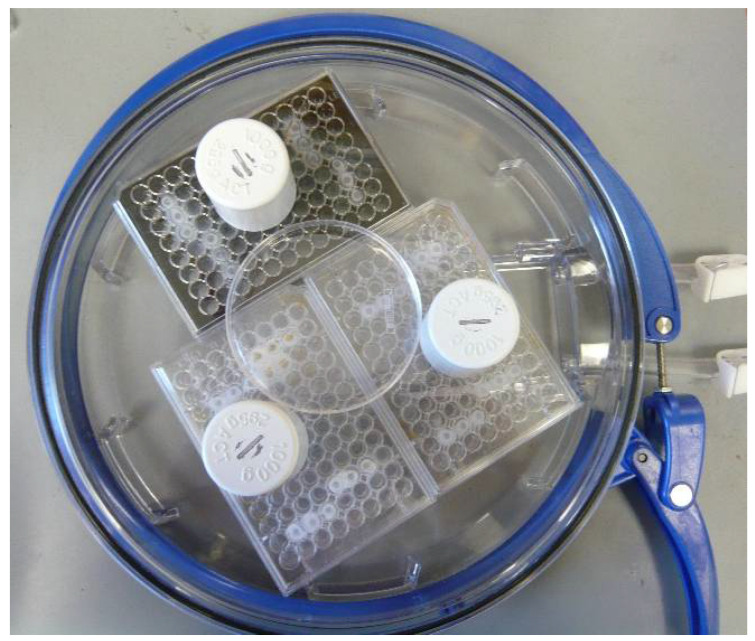
Image of compressed plates within the hypoxic chamber. Weights of 1000 g were placed on top of the self-constructed compression lids to apply a force of 9.81 N to an area of 0.079 cm^2^ per well.

We established an in vitro compression-type TBI model using MBEC, presented at international conferences [25,26]. MBEC were randomly divided into four groups: control/normoxia solely (CN-C), control/normoxia combined with compression (CN+C) mimicking contusion, hypoxia/reoxygenation without compression (HR-C) representing stroke and hypoxia/reoxygenation with compression (HR+C) simulating TBI (Figure 1). Normoxic conditions were represented by normal culturing conditions (complete media; 5% CO_2_, 21% O_2_; 74% N_2_, 37 °C). Hypoxic conditions were mimicked by exposure to glucose- and serum-free media, maintaining 0.01% O_2_, 5% CO_2_, 94.99% N_2_ at 37 °C within a hypoxic chamber (Stemcell Technologies; Vancouver, BC, Canada; Figure 2). Hypoxic/normoxic conditions were perpetuated for 5 or 15 h, representing mild-to-moderate or severe injury, respectively.

**Figure 3:**
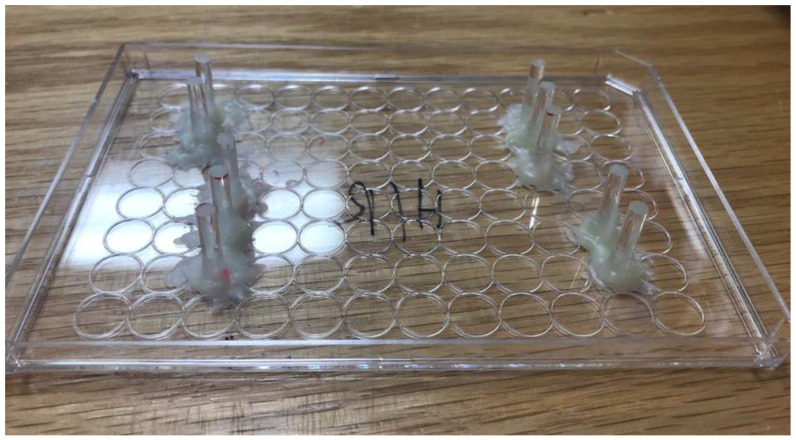
Image of the compression device. Plastic rods with 16 mm length and 3.175 mm diameter were fixed onto a lid of a 96-well plate.

Special lids (Figure 3) were constructed to compress an area of 0.079 cm^2^/well, using a force of 9.81 N. The application of compression was added for a timeframe of 1 h beginning upon hypoxia/normoxia.

Following hypoxia/normoxia the medium was replaced, and MBEC were cultured under regular culture conditions applying treatment with P188/PEG for 2 h to mimic reperfusion (Figure 2). Concentrations of 10 µM, 100 µM, and 1 mM of P188 (Sigma-Aldrich; St. Louis, MO, USA) or PEG (Sigma-Aldrich) dissolved in the medium were investigated for treatment effects measuring cell number/viability (V), cytotoxicity/membrane damage, metabolic activity (M) and total nitric oxide production (tNOp).

### 2.3. Cell Number/Viability

The cell number was determined using the fluorescence-based CyQUANT Direct Proliferation Assay Kit (Invitrogen; Carlsbad, CA, USA). The assay kit relies on a green fluorescent cell membrane permeable nucleic stain in combination with a cell membrane-impermeable background suppression dye, which prevents staining of dead cells or cells with the impaired cell membrane. As the fluorescence depends linearly on the cell number, it can be determined accurately. As instructed by the manufacturer, the 2X detection reagent was prepared, by combining PBS, CyQUANT Direct nucleic acid stain, and CyQUANT Direct background suppressor I within a light protected conical tube (for 12 mL of 2X detection reagent: 11.7 mL PBS (97.5%), 48 µL nucleic stain (0.4%) and 240 µL background suppressor I (2%)). The 2X detection reagent was mixed and stored at room temperature until utilization. Assay procedures were performed following 2 h of reoxygenation. Black 96-well plates were washed with 10% PBS twice, 50 µL of complete MBEC media (Cell Biologics Inc.), and 50 µL of the previously prepared 2X detection reagent (resulting in a concentration of 50% 2X detection reagent) were added to each well. After tapping gently, the cells were incubated at 37 °C for approximately 60 min. Afterward, the fluorescence was read with a plate reader (Synergy H1, BioTek Instruments Inc., Winooski, VT, USA) using an excitation wavelength of 480 nm and an emission wavelength of 535 nm (bottom read).

### 2.4. Cytotoxicity/Membrane Damage via Lactate Dehydrogenase Release

Pierce Lactate Dehydrogenase (LDH) Cytotoxicity Assay Kit (Thermo Fisher Scientific; Waltham, MA, USA) was used to determine cytotoxicity/membrane damage. In healthy cells, LDH is a cytosolic enzyme. Following injury of the plasma membrane, LDH is released into the medium. We used a colorimetric method quantifying cellular cytotoxicity by measuring extracellular LDH, using a coupled enzymatic reaction. LDH catalyzes the reaction from lactate to pyruvate leading to a reduction of nicotinamide adenine dinucleotide from its oxidized (NAD^+^) to its reduced (NADH) form. The tetrazolium salt iodonitrotetrazolium was reduced to a red formazan product by diaphorase using NADH. The formazan formation is directly proportional to the amount of LDH release. This can be used to indicate the level of cytotoxicity/membrane disruption. The reagent was prepared and stored following the manufacturer’s instructions. Briefly, the vial containing the substrate mix (lyophilizate) was diluted with 11.4 mL ultrapure water. The assay buffer was thawed, while shielded from light. The reaction mix consists of 0.6 mL assay buffer (5%) and 11.4 mL substrate mix (95%). Firstly, 50 µL of the media of each well were transferred to a not pre-coated, clear 96-well plate. Afterward, 50 µL of the reaction mix (achieving a concentration of 50% reaction mix) was added, the plates were tapped gently and protected from light using aluminum foil. The plates were incubated at room temperature. After 30 min, 50 µL stop solution (concentration of approximately 33%) were added. Then, 10 min later, the absorbance within the media was measured at 490 nm using a plate reader (Synergy H1, BioTek Instruments Inc.). In a second step, 5 µL of lysis buffer (10X) were added to the original plate, containing the cells and 50 µL of residual media. The plates were incubated at 37 °C for 60 min and the assay was performed as described above. The absorbance (abs) was calculated:

abs(media)/(abs[media] + abs[lysed cells]).

### 2.5. Metabolic Activity

The metabolic activity was determined using the CellTiter 96 AQueous One Solution Cell Proliferation Assay (Promega; Madison, WI, USA). The tetrazolium compound (3- (4,5-dimethylthiazol-2-yl)-5-(3-carboxymethoxyphenyl)-2-(4-sulfophenyl)-2H-tetrazolium, inner salt; MTS) used in this assay is bio-reduced by metabolically active cells forming a colored formazan product, soluble in culture medium [28]. After thawing for 90 min at 20–25 °C, 20 µL of CellTiter 96 AQueous One Solution Reagent was added to each well containing 100 µL of media (achieving a concentration of approximately 16.67%). The plates were incubated in the cell culture incubator at 37 °C for 1–4 h in humified air with 5% CO_2_. The abs were read in the plate reader (Synergy H1, BioTek Instruments Inc.) at 490 nm. Lastly, the blank abs were subtracted from the total abs.

### 2.6. Total NO Production

The tNOp was assayed using the Cayman’s Nitrate/Nitrite Colorimetric Assay Kit (Cayman Chemical Company; Ann Arbor, MI, USA). Nitric Oxide (NO), a highly reactive, short-lived free radical, is synthesized in biological systems by enzymes of the NO synthase (NOS) family, including endothelial NOS (eNOS), neuronal NOS (nNOS), and inducible NOS (iNOS). Different cell types can produce NO, including endothelial cells. The main effect of NO is paracrine activation of guanylate cyclase, which leads to an intracellular increase in cyclic guanosine monophosphate causing smooth muscle cells to relax [29]. This assay measures the total production of NO by adding the amount of nitrite (NO_2_^−^) and the amount of nitrate (NO_3_^−^), which both represent the final products of NO. First the NO_3_^−^ is exposed to NO_3_^−^ reductase, converting it to NO_2_^−^. In a second step, the Griess Reagents are used to convert NO_2_^−^ to an azo chromophore. Measurement of the resulting abs can determine the NO_2_^−^ concentration. Reagents and samples were prepared and the assay was performed as instructed in the assay protocol. In short, 10 µL of enzyme cofactor mixture and nitrate reductase mixture were added to up to 80 µL samples (resulting concentrations: 10% enzyme cofactor mixture, 10% nitrate reductase mixture, 80% sample). After the required incubation time, 50 µL Griess Reagent R1 followed by 50 µL Griess Reagent R2 were supplemented (a concentration of 25% of each result). The abs were measured at 540 nm using a plate reader (Synergy H1, BioTek Instruments Inc.). A standard curve was prepared, following the instructions in the assay protocol, whenever the assay was performed.

### 2.7. Statistics

All data were analyzed following the same procedure using RStudio Team (2018; RStudio: Integrated Development for R. RStudio, Inc., Boston, MA, USA). The data was continuous and was normalized to the condition CN-C as control and calculated as percentages (expressed as decimals). First, data were tested for normality using the Shapiro–Wilk test and for homogeneity of variance via Levene’s test (passed when *p* > 0.05). Additionally, data were plotted as Q-Q- and residuals versus fits plots to suggest if the assumption of normal distribution and equal variances was reasonable. Logarithmic transformation and outlier correction was used to transform not normally distributed data to normal distribution. If the requirements (normal distribution, homogeneity of variance, and independent samples) were met, the groups were compared by parametric testing using one-way analysis of variance (ANOVA); non-parametric data sets were compared by Kruskal–Wallis test. If a significant difference among the groups was found, post-hoc comparisons of non-parametric data were conducted by Dunn’s (adjustment using bh method), of parametric data by Student–Newman–Keuls. If the sets of data were normally distributed, but not homoscedastic, Welch’s one-way ANOVA and post-hoc comparisons with Games–Howell tests were applied to data sets. Any differences were considered significant when *p* < 0.05 (two-tailed) and are labeled as brackets. To display data distribution accurately, all data (parametric and non-parametric) are graphically expressed as box plots.

## 3. Results

### 3.1. Effect of P188 after Mild-to-Moderate Injury

#### 3.1.1. Cell Number/Viability

**Figure 4:**
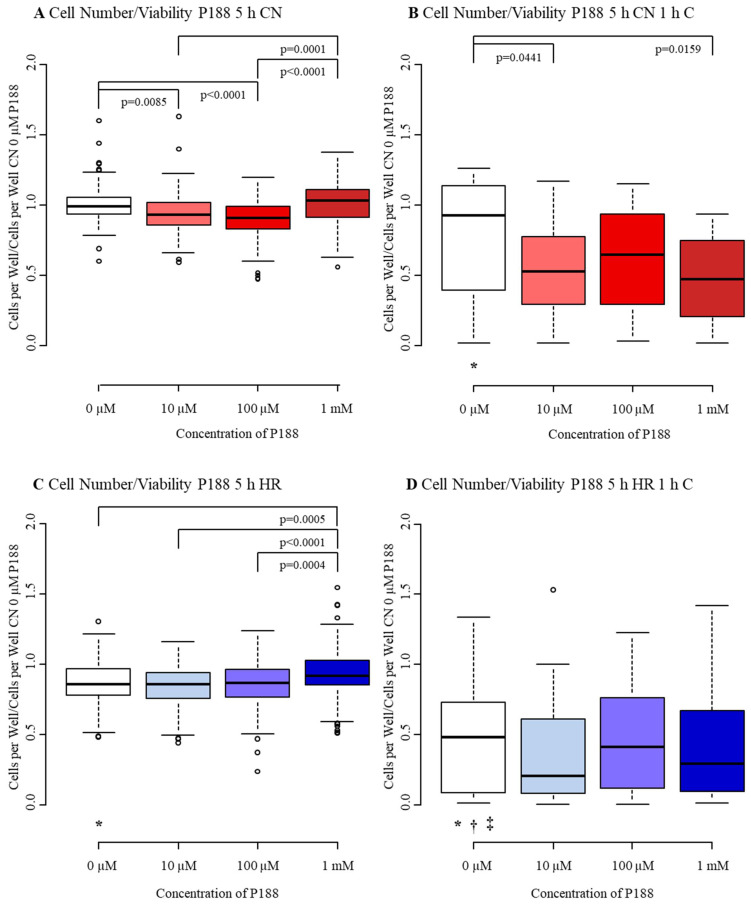
Cell number/viability after 5 h injury and treatment with 0 µM, 10 µM, 100 µM, and 1 mM of P188. (**A**) normoxia (CN-C); (**B**) normoxia & 1 h compression (CN+C); (**C**) hypoxia (HR-C); (**D**) hypoxia & 1 h compression (HR+C). Data visualized as boxplots (median, 25th, 75th percentile, whiskers: minimum, maximum, dots: potential outliers); *p* < 0.05; *n* = 12–19 experiments/group. Significances between different concentrations of P188 are displayed as brackets, significances between A, B, C, and D for 0 µM P188 are represented by symbols (* vs. CN-C 0 µM P188, † vs. CN+C 0 µM P188, ‡ vs. HR-C 0 µM P188). Abbreviations: C, compression; CN, control/normoxia; h, hours; HR, hypoxia/reoxygenation; mM, millimolar; µM, micromolar; P188, Poloxamer 188. Colors: red, normoxia; blue, hypoxia/reoxygenation. The shadows of red/blue represent the different concentrations of P188.

Control MBEC (CN-C) presented a median of 0.99 with an interquartile range from 0.93 to 1.06 in V. Exposure to compression-only (CN+C) and hypoxia only (HR-C) reduced V similarly by approximately 10% (CN+C: median 0.92, IQR 0.39–1.11; HR-C: median 0.86, IQR 0.78–0.97). When both were combined (HR+C), V was halved, displaying a median of 0.48 with an interquartile range from 0.09 to 0.73. While CN+C and HR-C injured MBEC mildly, HR+C damaged MBEC more severely (Figure 4A–D, Table A1).

Surprisingly, there was a slight decrease in V, when P188 was added to CN-C at low concentrations (10 µM, 100 µM), displaying medians of 0.93 and 0.91 and interquartile ranges from 0.86 to 1.02 and 0.83 to 0.99, respectively (Figure 4A). Consistently, V dropped when 10 µM and 1 mM P188 were applied to CN+C (Figure 4B). However, when added to HR-C, the presence of 1 mM P188 during reoxygenation improved V, nearly dividing the reduction of V following HR-C in half (Figure 4C). No differences in V were found when various concentrations of P188 were added to HR+C (Figure 4D).

To summarize, high concentrations of P188 given upon reoxygenation significantly increased the cell number of hypoxic cells.

#### 3.1.2. Cytotoxicity/Membrane Damage via LDH Release

**Figure 5:**
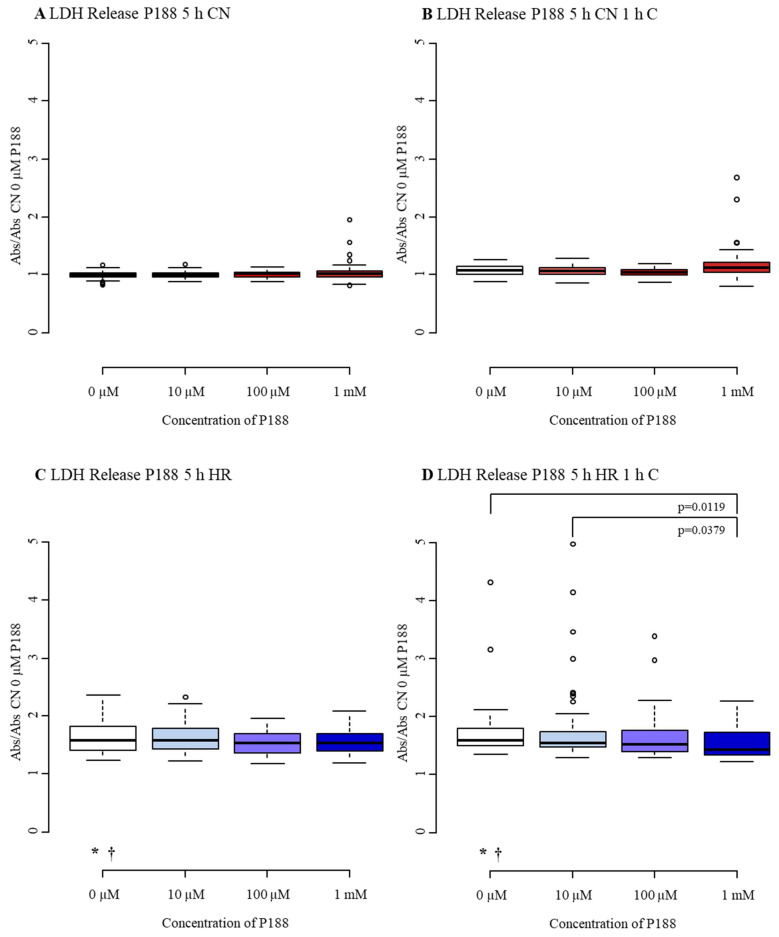
Cytotoxicity/membrane damage after 5 h injury and treatment with 0 µM, 10 µM, 100 µM, and 1 mM of P188. (**A**) normoxia (CN-C); (**B**) normoxia and 1 h compression (CN+C); (**C**) hypoxia (HR-C); (**D**) hypoxia and 1 h compression (HR+C). Data visualized as boxplots (median, 25th, 75th percentile, whiskers: minimum, maximum, dots: potential outliers); *p* < 0.05; *n* = 12–18 experiments/group. Significances between different concentrations of P188 are displayed as brackets, significances between A, B, C, and D for 0 µM P188 are represented by symbols (* vs. CN-C 0 µM P188, † vs. CN+C 0 µM P188). Abbreviations: Abs, absorbance; C, compression; CN, control/normoxia; h, hours; HR, hypoxia/reoxygenation; LDH, lactate dehydrogenase; mM, millimolar; µM, micromolar; P188, Poloxamer 188. Colors: red, normoxia; blue, hypoxia/reoxygenation. The shadows of red/blue represent the different concentrations of P188.

LDH release of CN-C MBEC showed a median of 1.00 with an interquartile range from 0.97 to 1.03. Interestingly, compression was not able to enhance LDH release, as CN+C did not differ from CN-C and HR-C was indifferent from HR+C. However, exposure to hypoxic conditions increased LDH release by nearly 60% compared to CN-C, with a median of 1.58 and an interquartile range from 1.41 to 1.82 (Figure 5A–D, Table A2).

No differences in LDH release became apparent with various concentrations of P188 treatment in CN-C, CN+C and HR-C (Figure 5A–C). Nevertheless, LDH release following HR+C was reduced by 14% when 1 mM P188 was present during reoxygenation compared to no treatment (Figure 5D). Collectively, 1 mM P188 given upon reoxygenation, inhibited rise in cytotoxicity in response to HR+C injury.

#### 3.1.3. Metabolic Activity

**Figure 6:**
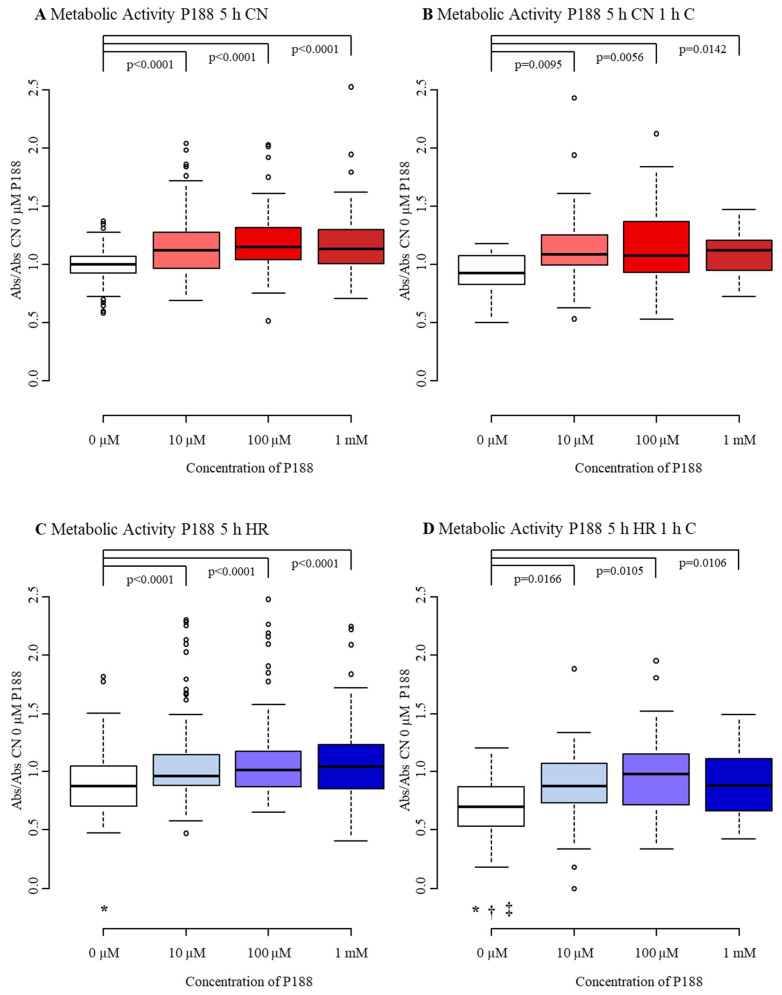
Metabolic activity after 5 h injury and treatment with 0 µM, 10 µM, 100 µM, and 1 mM of P188. (**A**) normoxia (CN-C); (**B**) normoxia and 1 h compression (CN+C); (**C**) hypoxia (HR-C); (**D**) hypoxia and 1 h compression (HR+C). Data visualized as boxplots (median, 25th, 75th percentile, whiskers: minimum, maximum, dots: potential outliers); *p* < 0.05; *n* = 10–19 experiments/group. Significances between different concentrations of P188 are displayed as brackets, significances between A, B, C, and D for 0 µM P188 are represented by symbols (* vs. CN-C 0 µM P188, † vs. CN+C 0 µM P188, ‡ vs. HR-C 0 µM P188). Abbreviations: Abs, absorbance; C, compression; CN, control/normoxia; h, hours; HR, hypoxia/reoxygenation; mM, millimolar; µM, micromolar; P188, Poloxamer 188. Colors: red, normoxia; blue, hypoxia/reoxygenation. The shadows of red/blue represent the different concentrations of P188.

CN-C displayed a median M of 1.00 with an interquartile range from 0.93 to 1.07. Addition of compression only was not able to attenuate M of MBEC. Nevertheless, exposure to hypoxic conditions (HR-C) reduced M by about 13%, with a median of 0.87 and an interquartile range from 0.71 to 1.05. Similarly to V, the combination of hypoxia and compression (HR+C) was able to decrease M further, by decreasing M by a third, with a median of 0.70 and an interquartile range of 0.55 to 0.86. While compression alone did not affect M, exposure to hypoxia lessened M, and the combination of both additionally damaged MBEC (Figure 6A–D, Table A3).

M, regardless of treatment (CN-C, CN+C, HR-C, HR+C), increased significantly by adding 10 µM, 100 µM and 1 mM P188, except for 1 mM P188 in HR+C where an increase seemed to occur, but failed to reach significance. Higher concentrations of P188 did not show improved M compared to lower concentrations. The greatest increase in M was seen in CN-C when 100 µM P188 were applied, with a median of 1.15 and an interquartile range from 1.04 to 1.32 (Figure 6A). Comparable rises in M approaching 20% were observed in CN+C exposed MBEC when 10 µM and 100 µM of P188 were present. Precisely, the median of CN-C was 0.92 with an interquartile range from 0.83 to 1.08 (Figure 6B). The treatment groups had medians of 1.09, 1.08 and 1.12 for 10 µM, 100 µM and 1 mM P188, respectively. The largest elevation of M in HR-C MBEC was seen at 1 mM P188. With a median of 1.04 for 1 mM P188 and 1.02 for 100 µM P188 and interquartile ranges from 0.85 to 1.23 and 0.87 to 1.17, respectively, treatment restored M reaching control level (Figure 6C). HR+C showed comparable results, still the greatest increase in M was seen at a concentration of 100 µM P188, nearly leveling with CN-C with a median of 0.98 with an interquartile range from 0.72 to 1.15 (Figure 6D). P188 given upon reoxygenation elevated M in all treatment groups.

#### 3.1.4. Total NO Production

**Figure 7:**
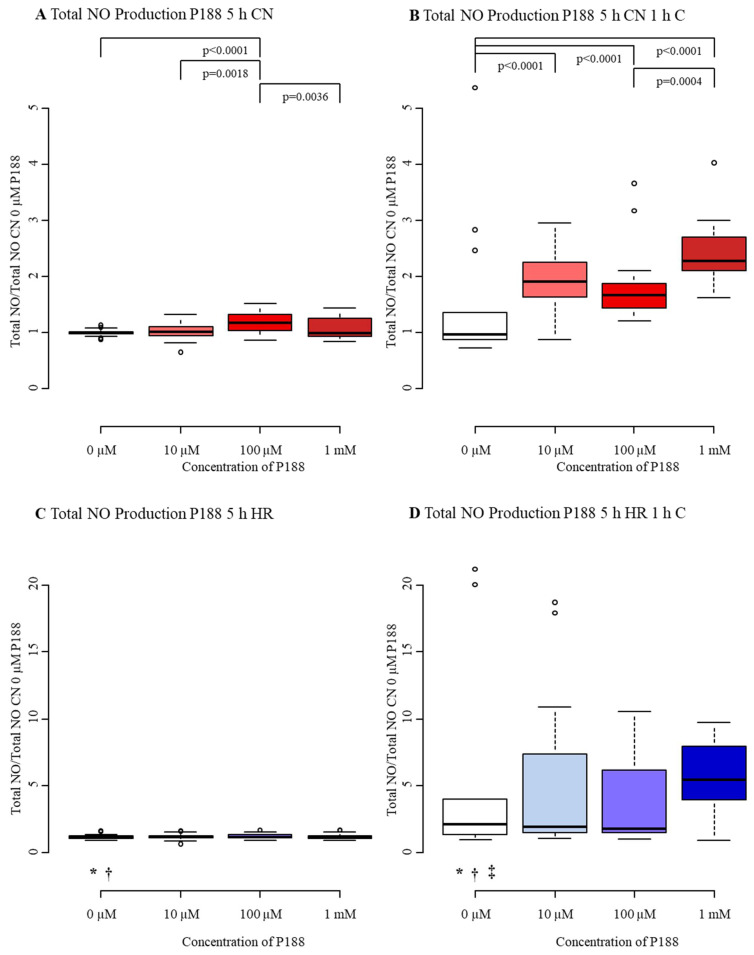
Total NO production after 5 h injury and treatment with 0 µM, 10 µM, 100 µM, and 1 mM of P188. (**A**) normoxia (CN-C); (**B**) normoxia and 1 h compression (CN+C); (**C**) hypoxia (HR-C); (**D**) hypoxia and 1 h compression (HR+C), two potential outliers were not displayed for esthetic reasons. Data visualized as boxplots (median, 25th, 75th percentile, whiskers: minimum, maximum, dots: potential outliers); *p* < 0.05; *n* = 8–10 experiments/group. Significances between different concentrations of P188 are displayed as brackets, significances between A, B, C, and D for 0 µM P188 are represented by symbols (* vs. CN-C 0 µM P188, † vs. CN+C 0 µM P188, ‡ vs. HR-C 0 µM P188). Abbreviations: C, compression; CN, control/normoxia; h, hours; HR, hypoxia/reoxygenation; mM, millimolar; µM, micromolar; NO, nitric oxide; P188, Poloxamer 188. Colors: red, normoxia; blue, hypoxia/reoxygenation. The shadows of red/blue represent the different concentrations of P188.

The median of tNOp in CN-C was 1.00 with an interquartile range from 0.98 to 1.02. When exposed to compression exclusively, the tNOp did not differ from control, whereas exposure to hypoxia only augmented tNOp, displayed by a median of 1.15 and an interquartile range from 1.07 to 1.25. The amalgamation of hypoxia and compression (HR+C) enhanced the increase potently up to a median of 2.10 accompanied by an interquartile range from 1.33 to 3.95 (Figure 7A–D, Table A4). In vitro TBI causes high levels of tNOp.

The role of NO in TBI is very complex and not fully understood yet. In our model, we observed an increase in tNOp following P188 treatment after exposure to CN-C and CN+C. The presence of 100 µM P188 during reoxygenation caused a 17% rise tNOp of CN-C MBEC (Figure 7A). Furthermore, all concentrations of P188 augmented tNOp of CN+C MBEC, with the largest increase at 1 mM P188, doubling tNOp (CN+C 1 mM P188: median 2.27, IQR 2.10–2.71; Figure 7B). In HR-C exposed MBEC no effect of P188 on tNOp became apparent (Figure 7C). Even though 1 mM P188 showed a trend to increase tNOp in HR+C exposed MBEC, this difference was not statistically significant (Figure 7D). Treatment with P188 seems to promote tNOp after injury.

Treatment with P188 after 5 h of simulated TBI enhanced the cell number, metabolic activity, and total NO production, while mounting cytotoxicity was inhibited.

### 3.2. Effect of P188 after Severe Injury

#### 3.2.1. Cell Number/Viability

**Figure A1:**
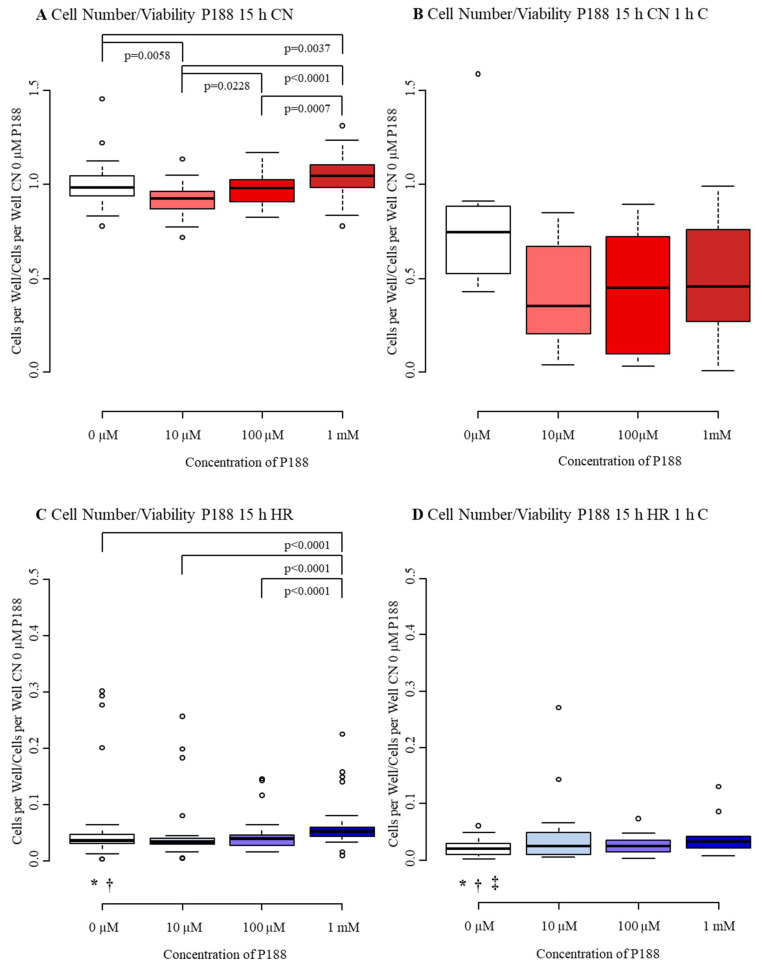
Cell number/viability after 15 h injury and treatment with 0 µM, 10 µM, 100 µM, and 1 mM of P188. (**A**) normoxia (CN-C); (**B**) normoxia and 1 h compression (CN+C); (**C**) hypoxia(HR-C); (**D**) hypoxia and 1 h compression (HR+C). Data visualized as boxplots (median, 25th, 75th percentile, whiskers: minimum, maximum, dots: potential outliers); *p* < 0.05; *n* = 4 experiments/group. Significances between different concentrations of P188 are displayed as brackets, significances between (**A**–**D**) for 0 µM P188 are represented by symbols (* vs. CN-C 0 µM P188, † vs. CN+C 0 µM P188, ‡ vs. HR-C 0 µM P188). Abbreviations: C, compression; CN, control/normoxia; h, hours; HR, hypoxia/reoxygenation; mM, millimolar; µM, micromolar; P188, Poloxamer 188. Colors: red, normoxia; blue, hypoxia/reoxygenation. The shadows of red/blue represent the different concentrations of P188.

Control MBEC (CN-C) had a median V of 0.98 with an interquartile range between 0.94 and 1.04. Compression applied for 1 h at the beginning of CN reduced V to approximately three-quarters. After 15 h of hypoxia and 2 h reoxygenation, V decreased excessively by 94%, the remaining V showed a median as small as 0.04 with an interquartile range from 0.03 to 0.05. The combination of hypoxia with compression (HR+C) decreased V even further, leaving only a median of 0.02 and an interquartile range between 0.01 and 0.03. Consequently, 1 h of compression had still an impact on V of MBEC after an additional 15 h of hypoxia. Moreover, 15 h hypoxia decreased V strongly, and HR+C even further (Figure A1A–D, Table A5).

While 10 µM P188 applied during CN-C exposure led to a significant decrease of 5% in V, adding 1 mM of P188 to CN-C increased V by approximately 6% (Figure A1A). Even though there was a non-significant trend of decreasing V when P188 was added, we did not find a significant difference between the concentrations of P188 in CN+C MBEC (Figure A1B). In HR-C MBEC treated with 1 mM of P188 upon reoxygenation, V increased slightly (Figure A1C). The other concentrations of P188 showed no effect. In HR+C cells the different concentrations of P188 did not change V (Figure A1D). While the highest concentration of P188 used seems to have a minuscule effect after extreme injury in normoxia-exposed and hypoxia-exposed MBEC, no effect was seen in compressed MBEC.

#### 3.2.2. Cytotoxicity/Membrane Damage via LDH Release

**Figure A2:**
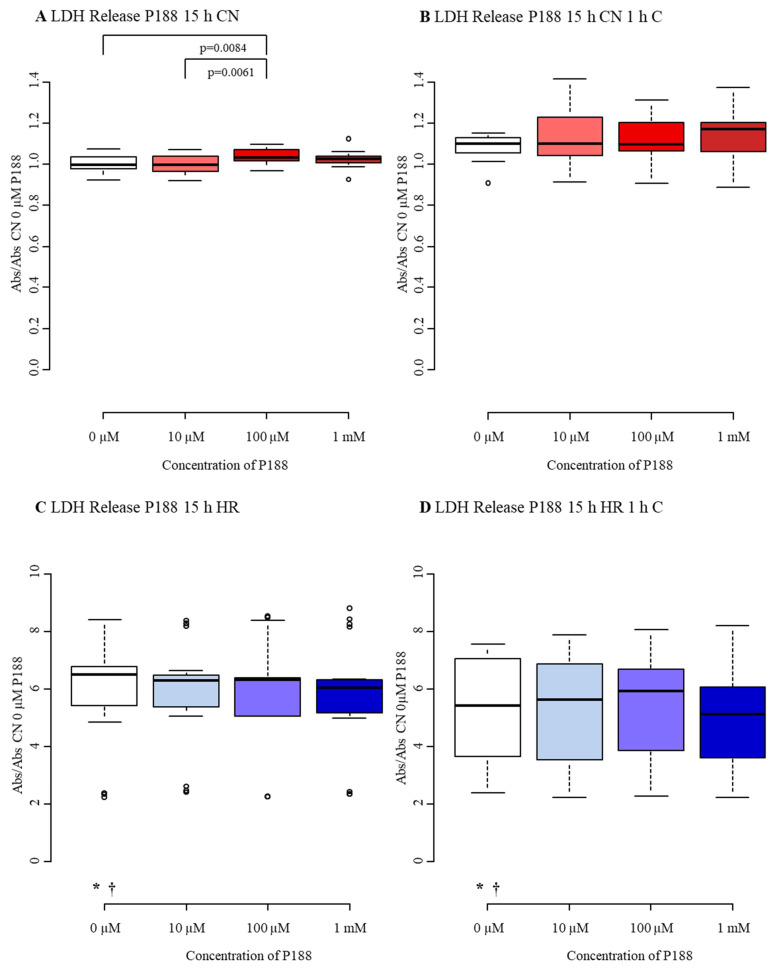
Cytotoxicity/membrane damage via LDH release after 15 h injury and treatment with 0 µM, 10 µM, 100 µM, and 1 mM of P188. (**A**) normoxia (CN-C); (**B**) normoxia and 1 h compression (CN+C); (**C**) hypoxia (HR-C); (**D**) hypoxia and 1 h compression (HR+C). Data visualized as boxplots (median, 25th, 75th percentile, whiskers: minimum, maximum, dots: potential outliers); *p* < 0.05; *n* = 4 experiments/group. Significances between different concentrations of P188 are displayed as brackets, significances between (**A**–**D**) for 0 µM P188 are represented by symbols (* vs. CN-C 0 µM P188, † vs. CN+C 0 µM P188). Abbreviations: Abs, absorbance; C, compression; CN, control/normoxia; h, hours; HR, hypoxia/reoxygenation; LDH, lactate dehydrogenase; mM, millimolar; µM, micromolar; P188, Poloxamer 188. Colors: red, normoxia; blue, hypoxia/reoxygenation. The shadows of red/blue represent the different concentrations of P188.

A six-fold increase in the release of LDH was observed from the control level with a median of 1.00 and an interquartile range from 0.98 to 1.04 to a median of 6.51 and an interquartile range between 5.41 and 6.76 following exposure to HR-C. Similar to LDH release in the 5 h injury model, hypoxia only caused dramatically mounting LDH release, while both compression groups (CN+C and HR+C) were not able to amplify LDH release further (Figure A2A–D, Table A6).

In CN-exposed MBEC, 100 µM P188 caused a 3% growth in LDH (Figure A2A). When compression was added to normoxia-exposed MBEC, no differences between the various concentrations of P188 were observed (Figure A2B). Comparably, no difference between the concentrations of P188 in HR-C MBEC was displayed, even though a non-significant trend towards reducing LDH release with a higher concentration of P188 became apparent (Figure A2C). Also, treatment with P188 was not able to protect MBEC from HR+C-related LDH release (Figure A2D).

#### 3.2.3. Metabolic Activity

**Figure A3:**
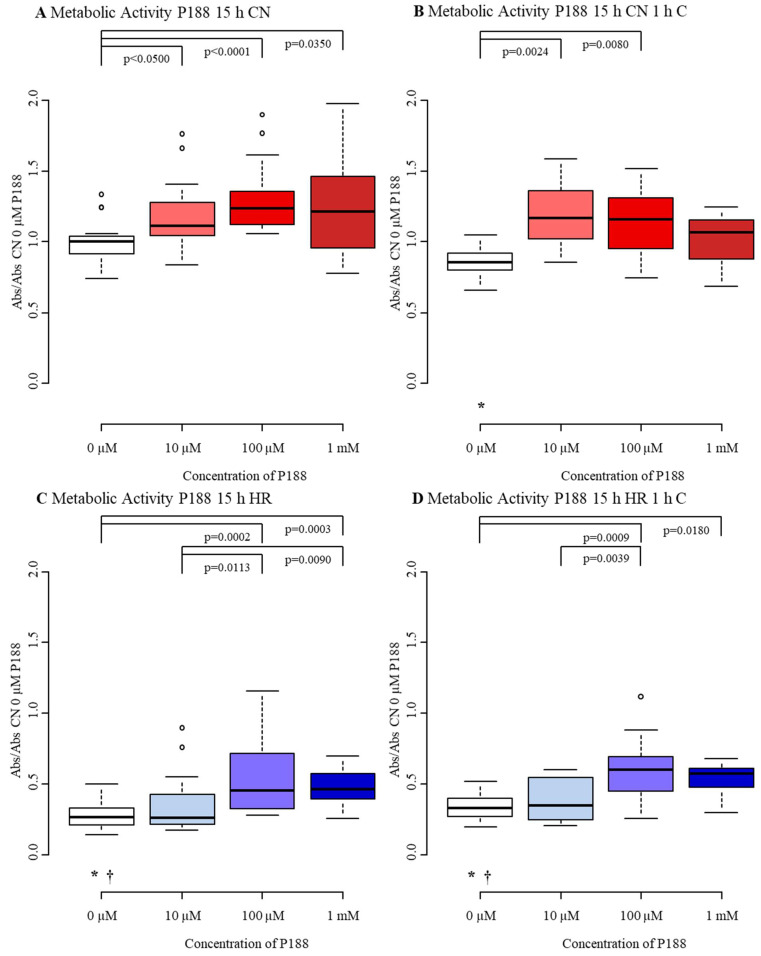
Metabolic activity after 15 h injury and treatment with 0 µM, 10 µM, 100 µM, and 1 mM of P188. (**A**) normoxia (CN-C); (**B**) normoxia and 1 h compression (CN+C); (**C**) hypoxia (HR-C); (**D**) hypoxia and 1 h compression (HR+C). Data visualized as boxplots (median, 25th, 75th percentile, whiskers: minimum, maximum, dots: potential outliers); *p* < 0.05; *n* = 4 experiments/group. Significances between different concentrations of P188 are displayed as brackets, significances between (**A**–**D**) for 0 µM P188 are represented by symbols (* vs. CN-C 0 µM P188, † vs. CN+C 0 µM P188). Abbreviations: Abs, absorbance; C, compression; CN, control/normoxia; h, hours; HR, hypoxia/reoxygenation; mM, millimolar; µM, micromolar; P188, Poloxamer 188. Colors: red, normoxia; blue, hypoxia/reoxygenation. The shadows of red/blue represent the different concentrations of P188.

The M of CN-C MBEC in the severe injury model displayed a median of 1.00 with an interquartile range from 0.92 to 1.04. Exposure to compression-only reduced M by 15%. 15 h of hypoxia diminished M dramatically, represented by a median of 0.27 and interquartile ranges between 0.21 and 0.33. The addition of 1 h compression to 15 h of hypoxia did not change M further than HR-C (Figure A3A–D, Table A7).

All different concentrations of P188 were able to enhance M in CN-C. The greatest increase (24%) appeared in the 100 µM P188 group, with a median of 1.24 and an interquartile range between 1.14 and 1.35 (Figure A3A). In CN+C MBEC, M was elevated by approximately 30% when 10 µM and 100 µM of P188 were added during reoxygenation compared to 0 µM P188 (Figure A3B). When 100 µM and 1 mM of P188 were present during reoxygenation in HR-C cells, a rise in M became apparent (Figure A3C). Similarly, M almost doubled when 100 µM and 1 mM of P188 were added to HR+C MBEC, with medians of 0.60 and 0.57, respectively (Figure A3D). To summarize, treatment with P188 improved metabolic activity after severe in vitro TBI in all treatment groups.

In this set of experiments, P188 was not able to show similar protective effects, as observed previously (cf. 3.1.). However, metabolic activity was still improved by P188.

### 3.3. Diminished Effect of PEG after Mild-to-Moderate Injury

#### 3.3.1. Cell Number/Viability

**Figure A4:**
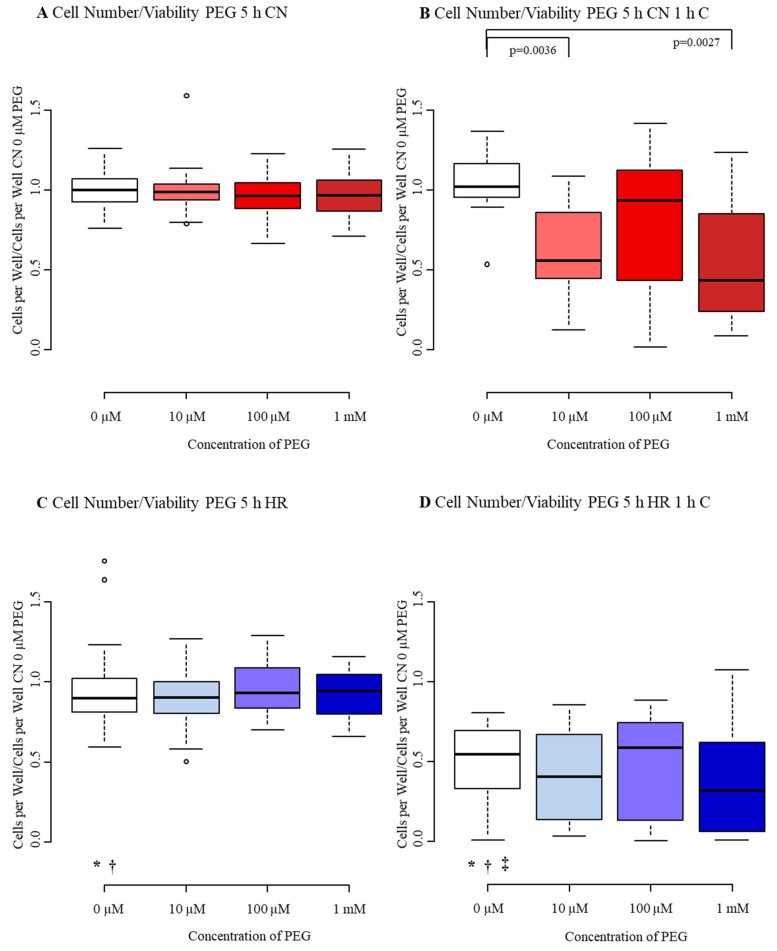
Cell number/viability after 5 h injury and treatment with 0 µM, 10 µM, 100 µM, and 1 mM of PEG. (**A**) normoxia (CN-C); (**B**) normoxia and 1 h compression (CN+C); (**C**) hypoxia (HR-C); (**D**) hypoxia and 1 h compression (HR+C). Data visualized as boxplots (median, 25th, 75th percentile, whiskers: minimum, maximum, dots: potential outliers); *p* < 0.05; *n* = 6–7 experiments/group. Significances between different concentrations of P188 are displayed as brackets, significances between (**A**–**D**) for 0 µM P188 are represented by symbols (* vs. CN-C 0 µM P188, † vs. CN+C 0 µM P188, ‡ vs. HR-C 0 µM P188). Abbreviations: C, compression; CN, control/normoxia; h, hours; HR, hypoxia/reoxygenation; mM, millimolar; µM, micromolar; PEG, polyethylene glycol. Colors: red, normoxia; blue, hypoxia/reoxygenation. The shadows of red/blue represent the different concentrations of PEG.

While V of CN+C did not differ from the CN-C, hypoxia solely reduced V by 10% and HR+C nearly cut V of CN-C in halve (Figure A4A–D, Table A8). Hypoxia only and combined with compression reduced V similarly to P188 (cf 3.1.1). However, CN-C did not display the same reduction.

No differences in V were found when comparing the various concentrations of PEG in CN-C, HR-C, and HR+C (Figure A4A,C,D). Only in CN+C-exposed MBEC was a difference seen, as the cell number was halved when 10 µM and 1 mM of PEG were added (Figure A4B). Treatment with PEG did not show the same benefit in V as P188. On the contrary, it even reduced V in compressed MBEC.

#### 3.3.2. Cytotoxicity/Membrane Damage via LDH Release

**Figure A5:**
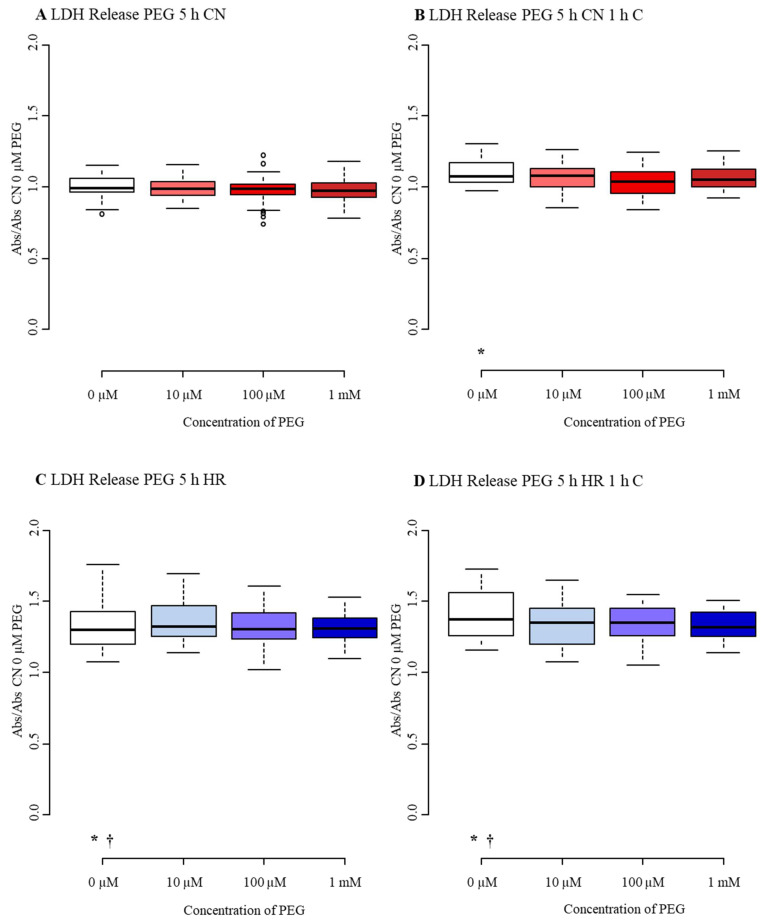
Cytotoxicity/membrane damage after 5 h injury and treatment with 0 µM, 10 µM, 100 µM, and 1 mM of PEG. (**A**) normoxia (CN-C); (**B**) normoxia and 1 h compression (CN+C); (**C**) hypoxia (HR-C); (**D**) hypoxia and 1 h compression (HR+C). Data visualized as boxplots (median, 25th, 75th percentile, whiskers: minimum, maximum, dots: potential outliers); *p* < 0.05; *n* = 6–7 experiments/group. Significances between different concentrations of P188 are displayed as brackets, significances between (**A**–**D**) for 0 µM P188 are represented by symbols (* vs. CN-C 0 µM P188, † vs. CN+C 0 µM P188). Abbreviations: Abs, absorbance; C, compression; CN, control/normoxia; h, hours; HR, hypoxia/reoxygenation; LDH, lactate dehydrogenase; mM, millimolar; µM, micromolar; PEG, polyethylene glycol. Colors: red, normoxia; blue, hypoxia/reoxygenation. The shadows of red/blue represent the different concentrations of PEG.

The median LDH release of control cells was 0.99 with an interquartile range from 0.97 to 1.06. Similar to the P188 group, CN+C did not affect LDH release (cf 3.1.2). However, HR+C did further increase LDH release of hypoxia only, resulting in a median of 1.45 and an interquartile range from 1.32 to 1.56 (Figure A5A–D, Table A9).

LDH release remained constant in all treatment groups (CN-C, CN+C, HR-C, HR+C) when 10 µM, 100 µM, and 1 mM PEG were added (Figure A5A–D). Interestingly, there was a slight non-significant trend towards lower LDH release in HR+C MBEC (Figure A5D). The positive effect on cytotoxicity by P188 was not observed in the PEG treatment group.

#### 3.3.3. Metabolic Activity

**Figure A6:**
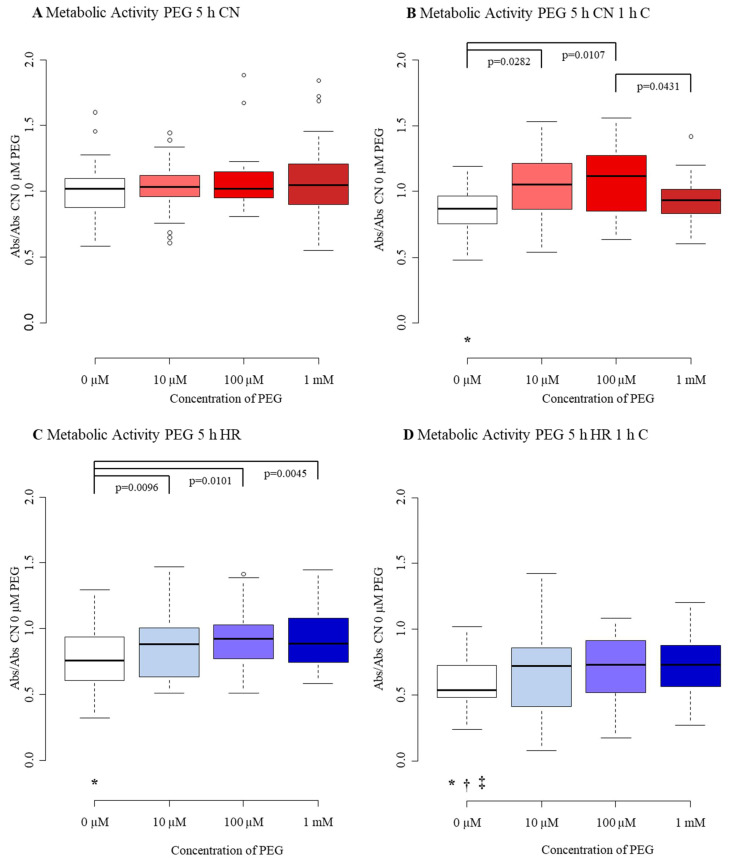
Metabolic activity after 5 h injury and treatment with 0 µM, 10 µM, 100 µM, and 1 mM of PEG. (**A**) normoxia (CN-C); (**B**) normoxia and 1 h compression (CN+C); (**C**) hypoxia (HR-C); (**D**) hypoxia and 1 h compression (HR+C). Data visualized as boxplots (median, 25th, 75th percentile, whiskers: minimum, maximum, dots: potential outliers); *p* < 0.05; *n* = 6–7 experiments/group. Significances between different concentrations of P188 are displayed as brackets, significances between (**A**–**D**) for 0 µM P188 are represented by symbols (* vs. CN-C 0 µM P188, † vs. CN+C 0 µM P188, ‡ vs. HR-C 0 µM P188). Abbreviations: Abs, absorbance; C, compression; CN, control/normoxia; h, hours; HR, hypoxia/reoxygenation; mM, millimolar; µM, micromolar; PEG, polyethylene glycol. Colors: red, normoxia; blue, hypoxia/reoxygenation. The shadows of red/blue represent the different concentrations of PEG.

Median M of CN-C MBEC was 1.02 with an interquartile range from 0.88 to 1.09. CN+C reduced M by 15%. The influence of hypoxia on M was greater, decreasing M by a quarter. The greatest decline was visible in the HR+C group, with a median of 0.54 and an interquartile range between 0.48 and 0.72 (Figure A6A–D, Table A10).

The response of M to treatment with PEG was variable. There was no significant difference, only a non-significant trend, between the concentrations of PEG when MBEC were exposed to CN-C (Figure A6A). Neither was there a significant difference between the different concentrations of PEG when cells were exposed to HR+C (Figure A6D). Nevertheless, M of CN+C exposed MBEC increased by 20% with 10 µM and by a quarter with 100 µM of PEG present during reoxygenation (Figure A6B). Comparably, M of hypoxia-exposed MBEC climbed with all concentrations of PEG with the greatest increase at 1 mM PEG, almost reaching the level of control (Figure A6C). PEG seems to influence M of MBEC to some degree.

## 4. Discussion

TBI represents a major health concern, as it contributes largely to disability and death [30]. Yet, not only TBI itself, but mostly downstream cascades taking place as a consequence of the impact can severely injure the brain tissue [2,31]. Even though the primary injury can severely impair neurons, glia, and cerebral blood vessels in multiple ways, including cerebral hemorrhages, contusions, and traumatic axonal injury [2], it can only be positively affected through prevention [4]. Nevertheless, therapies specifically targeting secondary injury are not yet established [2,6]. Ischemia is thought to play a central role in evolving secondary brain injury. Even though perfusion needs to be restored, reperfusion can enhance ischemic damage. Besides myocardial infarction, I/R injury was found in many organs, e.g., as a result of stroke and TBI [7]. Previously, we were able to show, that our specialists in vitro approach, combining 5 h hypoxia and 1 h compression, followed by 2 h reoxygenation, is suitable to simulate TBI and test for potential treatments [25,26]. Additionally, our model can mimic contusion (CN+C) and stroke (HR-C).

In this study, 5 h of hypoxia combined with compression applied mild to moderate damage to MBEC by reducing cell number/viability and metabolic activity, while increasing LDH release and total NO production. In detail, when examining 1 h compression during a period of 5 h normoxia followed by 2 h reoxygenation, we observed only small damage, as we did not see a difference to normoxia-exposed cells in total NO production. Moreover, as the injury was very small, the results in LDH release, metabolic activity, and cell number/viability varied from a small, but a significant injury to not significant results. This implies that concordant with Meyer et al., compression administered during normoxic conditions injures the cell inconsistently. Consequently, not every assay and set of data displayed similar significances [27]. However, the effect of hypoxia was consistent through all groups and assays, as the cell number/viability, metabolic activity, LDH release, and total NO production of hypoxia exposed MBEC was significantly lower than the values of normoxia-exposed cells. Similarly, the extent of the injury after hypoxia combined with compression was different from control in cell number/viability, metabolic activity, LDH release, and total NO production. However, compression was not consistently able to further amplify the amount of injury produced by hypoxia alone. While the combination of hypoxia and compression did enhance cellular damage observed after hypoxia alone in cell number/viability, metabolic activity, and total NO production, we were not able to observe the same effect for LDH release.

Additional to 5 h hypoxia, we also applied hypoxia for 15 h, while keeping the time of compression constant. In this set of experiments, we evaluated 15 h of hypoxia as extreme damage, to study the potential effects of treatment in severely damaged cells. Here, we found that compression administered at the beginning of a 15 h period of normoxia reduced cell number/viability and metabolic activity compared to control. The long duration of hypoxic conditions was able to powerfully reduce cell number/viability and metabolic activity while increasing LDH release. Similarly, hypoxia combined with compression decreased cell number/viability and metabolic activity and enhanced LDH release compared to normoxic cells. Still, compression was only able to further accelerate injury sustained by exposure to hypoxia in cell number/viability, not in LDH release and metabolic activity. A possible explanation might be that 15 h of hypoxia alone already caused a massive release of LDH and reduction in metabolic activity, not allowing further enhancement through the addition of short-lived compression. However, as the additional effect of compression in hypoxic cells was also not visible in LDH release after 5 h, it is possible that compression did not affect LDH release appropriately in MBEC. The results of Meyer et al. indicate that this cannot be transferred to neurons, suggesting an endothelial response to this kind of injury [27].

Based on these results, the model was utilized to apply treatment and to test the hypothesis that P188 exerts its protective effect in TBI and I/R injury through endothelial stabilization. P188 protects various cell types, including coronary endothelial cells, from I/R injury [13,14,21,32]. Moreover, P188 was found to have a beneficial effect in simulated stroke, TBI, and hemorrhagic injury models conducted in vitro [15,16,17,18,19,20,33,34,35] and in vivo [21,23,36,37]. Furthermore, P188 protected cells against excitotoxic injury [38,39,40,41]. Recently, Meyer et al. conducted a study with primary neuronal cell cultures applying a similar injury mechanism as in this protocol but failing to find a neuroprotective effect of P188 [27]. However, P188 does also influence other cell types than neurons. For example, implantation of an electrode covered with P188 reduced inflammation around the implanted device, compared to no cover [42]. Similarly, Zhang et al. described an anti-inflammatory effect of P188 on microglia/macrophages and astrocytes [36]. Interestingly, P188 was also anti-inflammatory in a dopaminergic neuron degeneration model [43]. These studies implicate that the neuroprotective effect of P188 exceeds direct neuronal protection. This is further strengthened by the direct protective effect of P188 on astrocytes in vitro [20]. Besides the effect on inflammatory cells, P188 was able to be advantageous in an in vitro blast-TBI model using primary brain microvascular endothelial cells [19], emphasizing a possible role of endothelial cells in neuroprotection. Furthermore, the BBB is thought to be of major importance in the development of TBI [44]. Besides improved cerebral blood flow in vivo following P188 treatment [45], P188 may have the ability to restore harmed plasma membranes of all cells comprising the neurovascular unit [23], which in fact may contribute majorly to its protective mechanism [24]. Therefore, we aimed to investigate whether P188 also exerts its protective effect in TBI and I/R injury through endothelial stabilization.

In another set of experiments, we chose to administer the hydrophobic polymer PEG with a molecular weight of 8000 Da [46] at the same concentrations as P188 before. With a molecular weight comparable to the molecular weight of P188 [47] and similar osmotic effects [13], PEG is the optimal polymer to assess whether P188 executes protection through its amphiphilic character or through its osmotic properties.

### 4.1. Cell Number/Viability

In CN-C-exposed MBEC, we found a slight decrease in cell number/viability in presence of low concentrations P188. After 15 h normoxia, cell number/viability reduced marginally with the application of 10 µM P188, while increasing with 1 mM P188 present. These findings are surprising, as direct observational studies of P188 membrane insertion were able to show that P188 is only integrated into lipid membranes when local lipid packing density is impaired. This suggests that P188 is only able to interact with injured lipid bilayers while being squeezed out of intact membranes, due to restored surface pressure [48,49]. Similarly, Wang et al. visualized P188 using α-amino-ω-BODIPY in an oxygen-glucose deprivation model. They found only a few P188 molecules in control embryonic hippocampal neurons [11]. Based on these findings, it would seem likely that P188 does not affect uninjured healthy cells. Correspondingly, many studies found no effect on different control cell types, including neurons [18] and cardiomyocytes [13]. Also, P188 revealed no toxicity in an assay based on 3-(4,5-dimethylthiazolyl-2)-2,5- diphenyltetrazolium bromide, when administered for 24, 48, and 72 h to a corneal endothelial cell line [50]. In contrast, some studies were able to observe an effect of P188 on sham cells. The baseline production of reactive oxygen species (ROS) increased in astrocytes upon treatment with P188, while P188 decreased ROS production after injury [20]. Similarly, the cell viability of human neuroblastoma cells decreased when treated with high concentrations of P188, although no effect was seen at low concentrations [51]. Furthermore, hippocampal cells lost cellular viability in normal conditions, when presented to 10^−4^ and 10^−3^ M P188 [21]. Interestingly, a study on primary brain microvascular endothelial cells observed no effect of P188 on cell viability up until 0.5 mM P188; however, when 750 µM P188 were administered cell viability decreased [19]. To summarize, the effect of P188 on control endothelial cells seems to be complex and may be connected to the concentrations of P188 applied. Even though literature implies that high concentrations affect cellular viability [19,21,51], we also observed adverse effects with low concentrations of P188. As the membrane sealing effect only seems to occur in injured neurons [48], one could suggest that P188 exhibits additional effects through different mechanisms. However, further studies need to be conducted to explicitly observe the effect of P188 on sham endothelial cells.

After exposure to I/R injury, we were able to show a protective effect of P188 on cell number/viability in our model applying mild-to-moderate damage. Contrary to our results, Meyer et al. did not see an improvement in cell number/viability after I/R injury of neurons [27]. Moreover, P188 did not show an effect on histological scores after spinal cord injury applied through occlusion of the thoracic aorta and subclavian arteries for 13 min [52]. Then again, P188 was able to enhance the survival of hippocampal neurons in an oxygen-glucose deprivation model [35]. Similarly, injury through 3 h glucose deprivation and hypoxia followed by normoxia, was able to mitigate cell death [34]. Also, Salzman et al. were able to show an increase in cell number of coronary artery endothelial cells, when P188 was administered upon reoxygenation [14]. Lastly, P188 was described to protect against I/R injury in vivo and in vitro, including protection of the BBB [21].

All in all, our findings of a protective effect of P188 on endothelial cells in vitro by increasing cell number are consistent with findings of previous studies. Moreover, we did not see a similar effect on endothelial cells when PEG was administered, suggesting that the protective mechanism occurs due to the unique amphiphilic character of P188 rather than its osmotic effects. Comparable results were described by Meyer et al. and Salzman et al., where PEG did not protect neurons and cardiomyocytes from TBI and I/R injury [13,27].

### 4.2. Cytotoxicity/Membrane Damage

On one hand, our results demonstrate a decrease of LDH release following treatment with P188 in hypoxia-exposed and compressed MBEC. On the other hand, we did not see the same effect when only one of the injury mechanisms was applied. Moreover, LDH release increased slightly with treatment after 15 h of normoxia. While a possible explanation for the rise in LDH release is the conflicting effect of P188 on control cells evaluated above, we would have expected to see a decrease in LDH release in all injured groups. However, the missing effect of P188 on compressed MBEC has to be interpreted cautiously as the harm on endothelial cells was not consistent in this group and the amount of damage could have been too inconsistent to show beneficial results. Still, in the literature, P188 was able to attenuate LDH release after hypoxic injury. For example, LDH release of coronary artery endothelial cells was decreased after I/R injury following P188 treatment [14]. Additionally, Martindale et al. described inhibition of LDH release in cardiomyocytes in response to copolymer-based sarcolemmal stabilization after I/R injury [53]. Similarly, in an ischemic model using neurons, LDH release was mitigated by P188 [34]. However, Meyer et al. did not find the same effect on LDH release in neurons after exposure to hypoxia or after exposure to hypoxia and compression [27]. Besides the lacking effect of P188 on hypoxia-exposed MBEC, we were able to show protection against HR+C. While this is different from the findings of Meyer et al. [27], a reduction in LDH release of neuronal cells ensuing P188 treatment was presented previously in shear stress models [15,18]. Even though we were able to show a reduction in LDH release in HR+C, we were not able to replicate the results of I/R injury studies. Concerning treatment with PEG after injury, we did not find an effect on LDH release. These findings are consistent with previous studies, as PEG was not able to protect cardiomyocytes and neurons from LDH release following simulated TBI or I/R [13,27,53].

### 4.3. Metabolic Activity

When assessing metabolic activity, we were able to see a beneficial effect of P188 in nearly all groups, including control and injured MBEC. To place these results into context, it is important to consider that we used the CellTiter 96 AQueous One Solution Cell Proliferation Assay which uses the bioreduction of MTS in metabolically active cells forming a colored formazan product, soluble in culture medium [28]. This assay can also be used to assess cellular number, viability, and mitochondrial viability, which it was aimed to do in previous studies [13,27,54,55]. However, as the CyQUANT Direct Proliferation Assay Kit, we used to measure cell number/viability does not require active metabolism [56], while the CellTiter 96 AQueous One Solution Cell Proliferation Assay depends on bioreduction [57], we used CellTiter 96 AQueous One Solution Cell Proliferation Assay to measure metabolic activity. Only a few studies have previously used MTS to evaluate the effect of P188 in TBI or I/R injury. While Salzman et al. [13] found an increase in cell number/viability, assessed with MTS, in hypoxia-exposed cardiomyocytes, Meyer et al. [27] only found a slight increase in one group. However, assessment of viability via a similar assay based on 3-(4,5-dimethylthiazol-2-yl)-2,5-diphenyltetrazolium bromide revealed a protective effect of P188 in shear stress models [15,18]. Interestingly, we also saw a rise in metabolic activity when CN+C- and HR-C-exposed MBEC were treated with PEG. This is contrary to our previous results where we were not able to see an effect of PEG. In vivo, Bartos et al. [58] showed that, while P188 reduced cellular and mitochondrial injury, PEG was not able to provide a benefit [58]. Concurrently, PEG did not enhance mitochondrial viability assessed by MTS in a neuronal TBI model [27]. Nevertheless, Marks et al. [38] observed the capacitance of adrenal chromaffin cells and found a momentarily amplified cell capacitance, still smaller than the effect of P188, when PEG with a similar molecular weight like P188 was applied. Our findings suggest that a small part of the beneficial effects of P188 on metabolic activity may be executed via osmotic properties.

### 4.4. Total NO Production

Furthermore, in our study, P188 increased tNOp in normoxic control cells as well as in compressed MBEC. Even though a climbing trend of tNOp was visible in HR+C exposed cells, this trend failed to achieve significance. In hypoxia-exposed MBEC, we were not able to see an effect of P188 on tNOp. Within the brain, NO increases cerebral perfusion and is used as a chemical messenger for inter-neuronal communication, synaptic plasticity, and neurotransmitter release [59]. Moreover, NO downregulates NMDAR [60] and reacts with ROS to form peroxynitrite [61]. Many factors contribute to NO having neuroprotective or neurotoxic properties [6]. The role of nitric oxide in TBI is quite complex, nevertheless, NO seems to play a part in the secondary injury mechanism after TBI [6,62]. NO can be synthesized by NOS, including eNOS, nNOS, and iNOS [29]. Neurons and endothelial cells produce NO shortly after injury. However, inflammatory cells produce NO as well at a later timepoint [6]. Interestingly, NO produced by nNOS seems to be disadvantageous on outcome after TBI, yet the NO produced by eNOS seems to be neuroprotective, as it ensures blood flow to the brain [59,63]. Douglas et al. proposed that the beneficial mechanism of P188 might be partly explained by enhancing NO release after detecting NO production in rat isolated hearts [64]. Similarly, Salzman et al. described, that the positive effects of P188 on infarct size and functional improvements were eliminated by administration of a nitric oxide synthase inhibitor [65]. Consistently, we found an increase in total NO production in MBEC, when P188 was added to compressed MBEC, suggesting that P188 might also exert neuroprotective effects via modulation of NO production. Numerous pathways are regulating eNOS activity in which P188 might interfere. One possible way to increase NO is simply by decreasing the activation of arginase. Traumatic brain injury is described to upregulate arginase activity, which competes with eNOS for the substrate L-arginine, causing a reduction in NO levels [66]. ROS can activate arginase [67], as Inyang et al. report a reduction of ROS by P188 [19] this might be one possible explanation for the rise in NO production. Another potential mechanism is based on the observation that P188 integrates into disrupted membranes sealing defects [12,22]. Hence, P188 could change the fluidity of the membrane and change the concentration of transmembrane proteins like caveolin-1. Caveolin-1 has been found to inhibit eNOS activity [68], P188 might change membrane properties influencing the membrane structure resulting in a lower concentration of caveolin-1 disinhibiting eNOS. However, the exact mechanism of eNOS activation by P188 should be addressed in future studies.

In conclusion, our model was able to demonstrate several directly protective effects of P188 on injured brain endothelial cells. These results suggest a protective role of P188 on endothelial cells, which might also contribute to the neuroprotection observed in vivo. However, the exact mechanism of P188 on endothelial cells and the role of P188 on the interaction between endothelial cells, neurons, and blood cells need to be explored in further detail.

### 4.5. Limitations

To allow an accurate review and discussion of our results, several limitations need to be acknowledged:

- We used an in vitro model which might not exactly mimic the complex pathogenesis of TBI in vivo. Morrison et al., though, suggested that in vitro models can predict effects in vivo in over 88% [69]. Even though 88% is quite high, some in vitro models do not adequately predict in vivo findings. However, in vitro models are reproducible, well-controlled, and can be performed within specific environments, without systemic confounders [69].
- MBEC were investigated. Even though mice are often used to mimic processes occurring in humans, they might not display the exact functioning of human brain microvascular endothelial cells. Song et al. found differences between mouse and human brain vasculature, that might be crucial for drug delivery and disease [70]. Interestingly, emerging approaches to address this limitation include an in vivo human blood vessel transplantation model [71].
- Although we were able to see significant effects of P188, some of these effects seem to be small in size, which might possibly reduce their relevance in preclinical studies. Nevertheless, we studied the effects on endothelial cells only, without taking into account that other cells of the neurovascular unit, neurons, and inflammatory cells may contribute to endothelial functioning and the effect of P188 in vivo. In addition, even small protective effects on endothelial cells might have amplified downstream effects on effector cells such as neurons as recently shown with cardiomyocytes [72].
- Furthermore, MBEC in the center of a well suffered more mechanical injury than cells growing at the margins of a well, as the rods of the compression devices were placed in the center. This might also have contributed to relatively high data variations observed in some sets of data.
- Another important limitation of this observational study is the absence of more detailed mechanistic experiments, which might further elucidate the underlying pathways of P188 protection in vitro in the future.
- Even though other hypoxia times might be suitable for MBEC [26], we only examined the effect of P188 on MBEC exposed to hypoxia for 5 and 15 h.
- Moreover, we did not observe the possible long-term effect of P188 administration. However, Gu et al. showed that daily intraperitoneal injection of P188 can enhance long-term outcomes after I/R injury [21]. In an in vitro blast-TBI model, endothelial cells were treated with P188 for 6 h with beneficial effects [19]. Consequently, the effect of P188 might differ when P188 is administered for a longer period than 2 h.
- Lastly, some of the protective effects of P188 have been described previously in various different models. However, the study by Inyang et al. [19] is the only one describing P188′s protective effect on isolated brain endothelial cells in the context of TBI. In contrast to our study that aimed to assess cellular functioning and cell survival after compression-injury in a hypoxia-reoxygenation model, though, they observed increased permeability, superoxide levels, and inflammatory trauma after blast-induced micro-cavitation, which included neither hypoxic nor reoxygenation injuries. Moreover, our study focused on very different endpoints, including but not limited to the effects of P188 on the production of NO which has not been described previously either and, thus, may address a possible mechanistic pathway for P188. Additionally, we are the first to show that the effect of P188 on MBEC when applied upon reoxygenation depends on the severity of the injury applied, with a decreasing effect after severe injury, limiting the protection by P188 to mild-to-moderate injury. Hence, our in vitro study does add several novel aspects to the current literature.

### 4.6. Future Directions

Future studies can build on the limitations of our model to expand these findings. Even though crucial properties of the BBB are mainly exerted by endothelial cells, BBB functioning relies on complex interactions between endothelial cells, pericytes, astrocytes, and immune cells [73]. As we were not able to evaluate possible interactions between adjacent cells, it would be interesting to observe effects on co-cultured endothelial cells. For example, astrocytes communicate with endothelial cells in multifaceted cell-cell contact influencing cellular physiology [74]. Moreover, there are approaches to co-culture endothelial cells with neurons to assess possible interactions [75]. Besides co-culturing three-dimensional (3D) culturing might be an elucidating approach. Our cell culturing only allows two-dimensional (2D) growth, nonetheless, this does not fully represent the 3D cellular expansion in vivo [76]. 2D culturing inhibits the possibility of cells to build tissue-like assemblies. However, 3D culturing allows cells to grow in a microenvironment allowing them to expand three-dimensionally and interact with surroundings as well as adjacent cells [76]. Captivatingly, Faley et al. already showed that induced pluripotent stem cell-derived human brain microvascular endothelial cells are capable to form confluent 3D monolayers [77], which makes 3D culturing even more attractive.

Additionally, rising attention is paid to diblock PEO-PPO polymers compared to the triblock P188. The structure of diblock polymers modifies the hydrophilic-lipophilic balance possible without changing the molecular weight [78]. Moreover, they have been shown to successfully stabilize impaired membranes in vivo [78]. Therefore, diblock copolymers have the potential to affect multiple chemical and biomedical applications, in compromised cell membranes [78].

## 5. Conclusions

In this in vitro study, the effect of P188 treatment upon reoxygenation was observed in simulated TBI, applying mild-to-moderate and severe damage. While we were able to see direct protective effects in mild-to-moderate in vitro TBI, supporting our hypothesis, only metabolic activity was influenced by P188 after severe injury. The exact mechanisms underlying endothelial protection and the influence of P188 on interactions with adjacent cells deserve to be further elucidated.

## Data Availability

Data are available from the authors upon reasonable request.

## Appendix B

**Table A1:** Cell number/viability after 5 h injury and treatment with 0 µM, 10 µM, 100 µM, and 1 mM of P188. Data from Figure 4 visualized as table (median, 25th, 75th percentile); *p* < 0.05; *n* = 12–19 experiments/group. Significances between different concentrations of P188 are displayed as superscripted numbers, significances between 0 µM P188 CN-C, CN+C, HR-C, and HR+C are represented by symbols (* vs. CN-C 0 µM P188, † vs. CN+C 0 µM P188, ‡ vs. HR-C 0 µM P188). Abbreviations: C, compression; CN, control/normoxia; h, hours; HR, hypoxia/reoxygenation; mM, millimolar; µM, micromolar; P188, Poloxamer 188.

Cell Number/Viability 5 h P188	0 µM P188 ^1^	10 µM P188 ^2^	100 µM P188 ^3^	1 mM P188 ^4^
CN-C	0.99 ^2,3^	0.93 ^1,4^	0.91 ^1,4^	1.03 ^2,3^
	(0.93, 1.06)	(0.86, 1.02)	(0.83, 0.99)	(0.91, 1.11)
CN+C	0.92 ^2,4,^*	0.53 ^1^	0.65	0.47 ^1^
	(0.39, 1.11)	(0.29, 0.77)	(0.30, 0.94)	(0.22, 0.74)
HR-C	0.86 ^4,^*	0.86 ^4^	0.87 ^4^	0.91 ^1,2,3^
	(0.78, 0.97)	(0.76, 0.94)	(0.76, 0.96)	(0.85, 1.03)
HR+C	0.48 *^,^^†,‡^	0.20	0.41	0.29
	(0.09, 0.73)	(0.09, 0.60)	(0.12, 0.76)	(0.09, 0.67)

**Table A2:** Cytotoxicity/membrane damage after 5 h injury and treatment with 0 µM, 10 µM, 100 µM, and 1 mM of P188. Data from Figure 5 visualized as table (median, 25th, 75th percentile); *p* < 0.05; *n* = 12–18 experiments/group. Significances between different concentrations of P188 are displayed as superscripted numbers, significances between 0 µM P188 CN-C, CN+C, HR-C, and HR+C are represented by symbols (* vs. CN-C 0 µM P188, † vs. CN+C 0 µM P188). Abbreviations: C, compression; CN, control/normoxia; h, hours; HR, hypoxia/reoxygenation; mM, millimolar; µM, micromolar; P188, Poloxamer 188.

LDH Release 5 h P188	0 µM P188 ^1^	10 µM P188 ^2^	100 µM P188 ^3^	1 mM P188 ^4^
CN-C	1.00	0.99	1.02	1.01
	(0.97, 1.03)	(0.97, 1.03)	(0.97, 1.05)	(0.97, 1.07)
CN+C	1.08	1.06	1.05	1.12
	(1.01, 1.14)	(1.01, 1.12)	(1.00, 1.09)	(1.04, 1.21)
HR-C	1.58 *^,†^	1.58	1.54	1.53
	(1.41, 1.82)	(1.44, 1.78)	(1.36, 1.70)	(1.40, 1.70)
HR+C	1.58 ^4,^*^,†^	1.55 ^4^	1.52	1.44 ^1,2^
	(1.50, 1.80)	(1.47, 1.74)	(1.40, 1.76)	(1.33, 1.72)

**Table A3:** Metabolic activity after 5 h injury and treatment with 0 µM, 10 µM, 100 µM, and 1 mM of P188. Data from Figure 6 visualized as table (median, 25th, 75th percentile); *p* < 0.05; *n* = 10–19 experiments/group. Significances between different concentrations of P188 are displayed as superscripted numbers, significances between 0 µM P188 CN-C, CN+C, HR-C, and HR+C are represented by symbols (* vs. CN-C 0 µM P188, † vs. CN+C 0 µM P188, ‡ vs. HR-C 0 µM P188). Abbreviations: C, compression; CN, control/normoxia; h, hours; HR, hypoxia/reoxygenation; mM, millimolar; µM, micromolar; P188, Poloxamer 188.

Mitochondrial Activity 5 h P188	0 µM P188 ^1^	10 µM P188 ^2^	100 µM P188 ^3^	1 mM P188 ^4^
CN-C	1.00 ^2,3,4^	1.12 ^1^	1.15 ^1^	1.13 ^1^
	(0.93, 1.07)	(0.97, 1.28)	(1.04, 1.32)	(1.01, 1.30)
CN+C	0.92 ^2,3,4^	1.09 ^1^	1.08 ^1^	1.12 ^1^
	(0.83, 1.08)	(1.00, 1.25)	(0.93, 1.36)	(0.95, 1.21)
HR-C	0.87 ^2,3,4,^*	0.96 ^1^	1.02 ^1^	1.04 ^1^
	(0.71, 1.05)	(0.88, 1.15)	(0.87, 1.17)	(0.85, 1.23)
HR+C	0.70 ^2,3,4,^ *^,^^†,‡^	0.88 ^1^	0.98 ^1^	0.88 ^1^
	(0.55, 0.86)	(0.73, 1.07)	(0.72, 1.15)	(0.68, 1.08)

**Table A4:** Total NO production after 5 h injury and treatment with 0 µM, 10 µM, 100 µM, and 1 mM of P188. Data from Figure 7 visualized as table (median, 25th, 75th percentile); *p* < 0.05; *n* = 8–10 experiments/group. Significances between different concentrations of P188 are displayed as superscripted numbers, significances between 0 µM P188 CN-C, CN+C, HR-C, and HR+C are represented by symbols (* vs. CN-C 0 µM P188, † vs. CN+C 0 µM P188, ‡ vs. HR-C 0 µM P188). Abbreviations: C, compression; CN, control/normoxia; h, hours; HR, hypoxia/reoxygenation; mM, millimolar; µM, micromolar; P188, Poloxamer 188.

Total NO Production 5 h P188	0 µM P188 ^1^	10 µM P188 ^2^	100 µM P188 ^3^	1 mM P188 ^4^
CN-C	1.00 ^3^	1.02 ^3^	1.17 ^4^	0.99 ^3^
	(0.98, 1.02)	(0.94, 1.10)	(1.04, 1.30)	(0.94, 1.25)
CN+C	0.97 ^2,3,4^	1.92 ^1^	1.67 ^1,4^	2.27 ^1,3^
	(0.89, 1.29)	(1.64, 2.17)	(1.45, 1.83)	(2.10, 2.71)
HR-C	1.15 *^,^^†^	1.19	1.15	1.20
	(1.07, 1.25)	(1.08, 1.28)	(1.08, 1.30)	(1.05, 1.28)
HR+C	2.10 *^,^^†,‡^	1.88	1.80	5.43
	(1.33, 3.95)	(1.45, 5.69)	(1.45, 6.17)	(3.99, 7.90)

**Table A5:** Cell number/viability after 15 h injury and treatment with 0 µM, 10 µM, 100 µM, and 1 mM of P188. Data from Figure A1 visualized as table (median, 25th, 75th percentile); *p* < 0.05; *n* = 4 experiments/group. Significances between different concentrations of P188 are displayed as superscripted numbers, significances between 0 µM P188 CN-C, CN+C, HR-C, and HR+C are represented by symbols (* vs. CN-C 0 µM P188, † vs. CN+C 0 µM P188, ‡ vs. HR-C 0 µM P188). Abbreviations: C, compression; CN, control/normoxia; h, hours; HR, hypoxia/reoxygenation; mM, millimolar; µM, micromolar; P188, Poloxamer 188.

Cell Number/Viability 15 h P188	0 µM P188 ^1^	10 µM P188 ^2^	100 µM P188 ^3^	1 mM P188 ^4^
CN-C	0.98 ^2,4^	0.93 ^1,3,4^	0.98 ^2,4^	1.04 ^1,2,3^
	(0.94, 1.04)	(0.88, 0.96)	(0.91, 1.02)	(0.98, 1.10)
CN+C	0.74	0.35	0.45	0.46
	(0.52, 0.88)	(0.23, 0.66)	(0.11, 0.72)	(0.27, 0.76)
HR-C	0.04 ^4,^*^,^^†^	0.03 ^4^	0.04 ^4^	0.05 ^1,2,3^
	(0.03, 0.05)	(0.03, 0.04)	(0.03, 0.05)	(0.04, 0.06)
HR+C	0.02 *^,^^†,‡^	0.02	0.02	0.03
	(0.01, 0.03)	(0.01, 0.04)	(0.02, 0.03)	(0.02, 0.04)

**Table A6:** Cytotoxicity/membrane damage after 15 h injury and treatment with 0 µM, 10 µM, 100 µM, and 1 mM of P188. Data from Figure A2 visualized as table (median, 25th, 75th percentile); *p* < 0.05; *n* = 4 experiments/group. Significances between different concentrations of P188 are displayed as superscripted numbers, significances between 0 µM P188 CN-C, CN+C, HR-C, and HR+C are represented by symbols (* vs. CN-C 0 µM P188, † vs. CN+C 0 µM P188). Abbreviations: C, compression; CN, control/normoxia; h, hours; HR, hypoxia/reoxygenation; mM, millimolar; µM, micromolar; P188, Poloxamer 188.

LDH Release 15 h P188	0 µM P188 ^1^	10 µM P188 ^2^	100 µM P188 ^3^	1 mM P188 ^4^
CN-C	1.00 ^3^	1.00^3^	1.03 ^1,2^	1.03
	(0.98, 1.04)	(0.97, 1.04)	(1.02, 1.07)	(1.01, 1.04)
CN+C	1.10	1.10	1.10	1.17
	(1.05, 1.13)	(1.05, 1.21)	(1.08, 1.19)	(1.06, 1.20)
HR-C	6.51 *^,^^†^	6.30	6.32	6.03
	(5.41, 6.76)	(5.38, 6.48)	(5.09, 6.40)	(5.16, 6.32)
HR+C	5.41 *^,^^†^	5.63	5.91	5.13
	(3.64, 7.05)	(4.14, 6.68)	(4.55, 6.65)	(3.59, 6.07)

**Table A7:** Metabolic activity after 15 h injury and treatment with 0 µM, 10 µM, 100 µM, and 1 mM of P188. Data from Figure A3 visualized as table (median, 25th, 75th percentile); *p* < 0.05; *n* = 4 experiments/group. Significances between different concentrations of P188 are displayed as superscripted numbers, significances between 0 µM P188 CN-C, CN+C, HR-C, and HR+C are represented by symbols (* vs. CN-C 0 µM P188, † vs. CN+C 0 µM P188). Abbreviations: C, compression; CN, control/normoxia; h, hours; HR, hypoxia/reoxygenation; mM, millimolar; µM, micromolar; P188, Poloxamer 188.

Metabolic Activity 15 h P188	0 µM P188 ^1^	10 µM P188 ^2^	100 µM P188 ^3^	1 mM P188 ^4^
CN-C	1.00 ^2,3,4^	1.11 ^1^	1.24 ^1^	1.21 ^1^
	(0.92, 1.04)	(1.05, 1.25)	(1.14, 1.35)	(0.99, 1.46)
CN+C	0.85 ^2,3,^*	1.17 ^1^	1.16 ^1^	1.07
	(0.80, 0.91)	(1.03, 1.34)	(0.96, 1.29)	(0.88, 1.15)
HR-C	0.27 ^3,4,^*^,^^†^	0.26 ^3,4^	0.45 ^1,2^	0.46 ^1,2^
	(0.21, 0.33)	(0.22, 0.42)	(0.33, 0.69)	(0.40, 0.57)
HR+C	0.33 ^3,4,^*^,^^†^	0.35 ^3^	0.60 ^1,2^	0.57 ^1^
	(0.27, 0.39)	(0.26, 0.53)	(0.50, 0.68)	(0.48, 0.61)

**Table A8:** Cell number/viability after 5 h injury and treatment with 0 µM, 10 µM, 100 µM, and 1 mM of PEG. Data from Figure A4 visualized as table (median, 25th, 75th percentile); *p* < 0.05; *n* = 6–7 experiments/group. Significances between different concentrations of PEG are displayed as superscripted numbers, significances between 0 µM PEG CN-C, CN+C, HR-C, and HR+C are represented by symbols (* vs. CN-C 0 µM PEG, † vs. CN+C 0 µM PEG, ‡ vs. HR-C 0 µM PEG). Abbreviations: C, compression; CN, control/normoxia; h, hours; HR, hypoxia/reoxygenation; mM, millimolar; µM, micromolar; PEG, polyethylene glycol.

Cell Number/Viability 5 h PEG	0 µM PEG ^1^	10 µM PEG ^2^	100 µM PEG ^3^	1 mM PEG ^4^
CN-C	1.00	0.99	0.96	0.97
	(0.93, 1.07)	(0.94, 1.04)	(0.88, 1.04)	(0.87, 1.06)
CN+C	1.02 ^2,4^	0.56 ^1^	0.93	0.43^1^
	(0.96, 1.14)	(0.46, 0.86)	(0.52, 1.12)	(0.24, 0.85)
HR-C	0.90 *^,^^†^	0.90	0.93	0.94
	(0.81, 1.03)	(0.80, 1.00)	(0.84, 1.09)	(0.80, 1.05)
HR+C	0.55 *^,^^†,‡^	0.40	0.58	0.32
	(0.33, 0.69)	(0.15, 0.67)	(1.16, 0.73)	(0.06, 0.62)

**Table A9:** Cytotoxicity/membrane damage after 5 h injury and treatment with 0 µM, 10 µM, 100 µM, and 1 mM of PEG. Data from Figure A5 visualized as table (median, 25th, 75th percentile); *p* < 0.05; *n* = 6–7 experiments/group. Significances between different concentrations of PEG are displayed as superscripted numbers, significances between 0 µM PEG CN-C, CN+C, HR-C, and HR+C are represented by symbols (* vs. CN-C 0 µM PEG, † vs. CN+C 0 µM PEG). Abbreviations: C, compression; CN, control/normoxia; h, hours; HR, hypoxia/reoxygenation; mM, millimolar; µM, micromolar; PEG, polyethylene glycol.

LDH 5 h PEG	0 µM PEG ^1^	10 µM PEG ^2^	100 µM PEG ^3^	1 mM PEG ^4^
CN-C	0.99	0.99	0.99	0.97
	(0.97, 1.06)	(0.94, 1.04)	(0.95, 1.02)	(0.93, 1.03)
CN+C	1.07 *	1.08	1.04	1.05
	(1.03, 1.17)	(1.00, 1.13)	(0.96, 1.10)	(1.00, 1.12)
HR-C	1.32 *^,^^†^	1.32	1.30	1.31
	(1.22, 1.42)	(1.25, 1.47)	(1.24, 1.41)	(1.24, 1.39)
HR+C	1.45 *^,^^†^	1.35	1.35	1.32
	(1.32, 1.56)	(1.20, 1.45)	(1.27, 1.44)	(1.25, 1.43)

**Table A10:** Metabolic activity after 5 h injury and treatment with 0 µM, 10 µM, 100 µM, and 1 mM of PEG. Data from Figure A6 visualized as table (median, 25th, 75th percentile); *p* < 0.05; *n* = 6–7 experiments/group. Significances between different concentrations of PEG are displayed as superscripted numbers, significances between 0 µM PEG CN-C, CN+C, HR-C, and HR+C are represented by symbols (* vs. CN-C 0 µM PEG, † vs. CN+C 0 µM PEG, ‡ vs. HR-C 0 µM PEG). Abbreviations: C, compression; CN, control/normoxia; h, hours; HR, hypoxia/reoxygenation; mM, millimolar; µM, micromolar; PEG, polyethylene glycol.

Metabolic Activity 5 h PEG	0 µM PEG ^1^	10 µM PEG ^2^	100 µM PEG ^3^	1 mM PEG ^4^
CN-C	1.02	1.03	1.02	1.05
	(0.88, 1.09)	(0.96, 1.12)	(0.95, 1.15)	(0.90, 1.21)
CN+C	0.87 ^2,3,^*	1.05 ^1^	1.12 ^1,4^	0.94 ^3^
	(0.77, 0.95)	(0.88, 1.20)	(0.87, 1.27)	(0.84, 1.02)
HR-C	0.75 ^2,3,4,^*	0.88 ^1^	0.92 ^1^	0.89 ^1^
	(0.60, 0.93)	(0.66, 1.00)	(0.77, 1.02)	(0.74, 1.08)
HR+C	0.54 *^,^^†,‡^	0.72	0.73	0.73
	(0.48, 0.72)	(0.45, 0.85)	(0.53, 0.90)	(0.56, 0.88)
